# Trajectories of maternal ante- and postpartum depressive symptoms and their association with child- and mother-related characteristics in a West African birth cohort study

**DOI:** 10.1371/journal.pone.0187267

**Published:** 2017-11-06

**Authors:** Dana Barthel, Levente Kriston, Daniel Fordjour, Yasmin Mohammed, Esther Doris Kra-Yao, Carine Esther Bony Kotchi, Ekissi Jean Koffi Armel, Kirsten Alexandra Eberhardt, Torsten Feldt, Rebecca Hinz, Koffi Mathurin, Stefanie Schoppen, Carola Bindt, Stephan Ehrhardt, on behalf of the International CDS Study Group

**Affiliations:** 1 Clinical Research Unit, Bernhard Nocht Institute for Tropical Medicine, Hamburg, Germany; 2 Department of Child and Adolescent Psychiatry, Psychotherapy, and Psychosomatics, University Medical Center Hamburg-Eppendorf, Hamburg, Germany; 3 Department of Medical Psychology, University Medical Center Hamburg-Eppendorf, Hamburg, Germany; 4 Department of Behavioural Sciences, School of Medical Sciences, Kwame Nkrumah University of Science and Technology, Kumasi, Ghana; 5 Laboratoire de Santé, Nutrition et Hygiène, Centre de Recherche pour le Développement, Université Alassane Ouattara, Bouaké, Côte d’Ivoire; 6 Département de Psychologie, Université Felix Houphouet Boigny de Cocody, Abidjan, Côte d’Ivoire; 7 Clinic of Gastroenterology, Hepatology and Infectious Diseases, Heinrich Heine University, Düsseldorf, Germany; 8 Institute of Medical Microbiology, Virology and Hygiene, University Medical Center Hamburg-Eppendorf, Germany; 9 Laboratoire des Interactions Hôte-Microorganisme, Jean Lorougnon Guede University, Daloa, Côte d’Ivoire; 10 Department of Epidemiology, Johns Hopkins Bloomberg School of Public Health, Baltimore, MD, United States of America; Southeast University Zhongda Hospital, CHINA

## Abstract

**Background:**

The vast majority of research on mental health has been undertaken in high income countries. This study aimed at investigating the long-term course of maternal depressive symptoms and its association with various mother- and child-related characteristics in two West African lower middle income countries with focus on the relationship with long-term anxiety symptoms.

**Methods:**

In the Child Development Study, a prospective birth cohort study in Côte d’Ivoire and Ghana, the 9-item Patient Health Questionnaire (PHQ-9) was answered by *N* = 776 women 3 months antepartum, and 3, 12, and 24 months postpartum between April 2010 and March 2014. Growth mixture modeling was used to identify distinct trajectories of depressive symptoms. Several psychosocial, obstetric, and sociodemographic characteristics were assessed and multinomial regression analysis was performed to investigate the influence of these variables on the different depression trajectories.

**Results:**

We found three distinct classes of depressive symptoms that were characterized by an asymptomatic trajectory (91.5%), by recurrent risk (4.3%) and by postnatal risk (4.3%). The longitudinal course of depressive symptoms was strongly associated with anxiety symptoms (*χ*^2^ = 258.54, df = 6, *p* < 0.001; *φ* = .577). Among other factors, higher levels of anxiety, new pregnancy 2 years after birth, economic stress, and family stress were associated with the risk classes.

**Conclusions:**

A substantial proportion of West African women in our sample developed unfavorable patterns of depressive symptoms during the vulnerable phase of pregnancy and early motherhood. Psychosocial factors, especially antepartum anxiety symptoms, played a decisive role in this process. Perceived economic hardship further exaggerated the mental health burden.

## Introduction

Ante- and postpartum maternal depression is a global public health problem [1, 2]. Besides the burden for the individual woman [3], perinatal depression may have adverse effects on the offspring’s health and development [4, 5], predicts their biological sensitivity to social stress well into adulthood [6], and may prevent them from achieving their developmental potential [7]. Consequently, the United Nations declared the improvement of maternal health, naturally including mental health aspects, in their Sustainable Development Goals [2, 8]. Women from low and middle income countries (LMIC) may be particularly disadvantaged in terms of mental health due to numerous stressors [9, 10]. Despite the great relevance particularly for women from LMIC, the vast majority of research has been conducted in countries of the Global North [11].

The classification of countries based on their national income per capita is provided by the World Bank which categorizes countries into low-income, lower middle-income, upper middle-income, and high income countries. The term LMIC contains low-income and lower middle-income countries. Africa is geographically divided into North Africa and sub-Saharan Africa. To the latter belong 49 of the 54 African countries, including all West African countries. All sub-Saharan countries (except South Africa), and therefore all West African countries are considered LMIC. The target population of this study are West African mothers, being part of the broader population of sub-Saharan Africa, which itself consists of several LMIC. Only a handful of studies have investigated the long-term course of depressive symptoms during pregnancy and after childbirth, none of which were performed in West Africa, or sub-Sahara or even in LMIC. Studies from HIC reported different findings on the course of perinatal depressive symptomatology. While Heron et al. [12] identified a stable and moderately improving global trajectory from the antepartum period to 8 months postpartum, Lee et al. [13] suggested a pattern with highest depression scores measured with the Hospital Anxiety and Depression Scale in the first and third trimesters of pregnancy and also a high prevalence of depressive symptoms assessed with the Edinburgh Postnatal Depression Scale six weeks postpartum. Martini et al. [14] and Mora et al. [15] found multiple longitudinal patterns of depressive symptoms, e.g. a large group of permanently asymptomatic women, frequent occurrence of symptoms after delivery, and general recovery from the ante- to the postpartum phase. Since there is a lack of evidence for women from LMIC, it is of great importance to gain more knowledge about different courses of perinatal depressive symptoms in order to decide on the proper timing for screening activities and preventive measures.

Besides the scarce evidence on trajectories of perinatal depressive symptoms, their relationships with potential risks and protective factors have been insufficiently studied, particularly in LMIC. Broadly, known correlates of perinatal depression can be grouped into psychosocial, obstetric, and sociodemographic factors.

Several psychosocial factors have been found to be associated with perinatal depressive symptoms. Findings from HIC suggest that both antepartum depression and antepartum anxiety, including a history of depression or anxiety disorders prior to pregnancy, might act as risk factors for postpartum depression [12–14]. Perinatal depression was also associated with a lower level of social support during the ante- and postpartum period [14], particularly with less partner support [16, 17]. Studies from LMIC showed that experience of both societal violence and intimate partner violence were associated with perinatal depressive symptoms [16, 18, 19]. Studies from both HIC and LMIC found that poor partnership satisfaction was associated with antepartum depressive symptoms [14, 19].

The effect of obstetric and sociodemographic factors on perinatal depression is less clear. Although some studies from LMIC suggested that nulliparous or primiparous women are at higher risk for depression [20, 21], others found the opposite [22, 23] or did not detect any association [24, 25]. Findings on the relationship between maternal age and perinatal depressive symptoms are also conflicting [17, 26]. More consistently, experiencing complications during previous pregnancies or deliveries, lower levels of education, and being single seem to increase the risk of perinatal depressive symptoms in LMIC [20, 21, 26, 27].

The aim of the present study was to investigate the long-term course of depressive symptoms in ante- and postpartum women in two West African lower middle income countries and to examine potential risk and protective factors associated with these trajectories, with a particular focus on the relationship with the longitudinal course of perinatal anxiety.

## Material and methods

### Study design

Data for this study were collected in the Child Development Study (CDS), which investigated a birth cohort in Côte d’Ivoire (CIV) and Ghana (GHA) including women and their children [28]. Women in their last trimester of pregnancy were consecutively recruited during antenatal care visits either at the Centre de Santé Urbain à base communautaire d’Abobo in Abidjan (CIV) or at the Komfo Anokye Teaching Hospital in Kumasi (GHA). The coverage of antepartum care is approximately 60% in CIV [29] and about 87% in Ghana [30]. Data were collected between April 2010 and March 2014. We used data from five measurement points for our analyses: recruitment (approximately 3 months before birth), birth, 3, 12 and 24 months after birth. All procedures were in accordance with the Helsinki declaration and the CDS was approved by the responsible ethical committees in each country, namely the national ethical committee in CIV, the ethical committee of the Kwame Nkrumah University of Science and Technology in Kumasi, Ghana, and the ethical committee of the chamber of physicians in Hamburg, Germany [28].

### Sample

**Fig 1:**
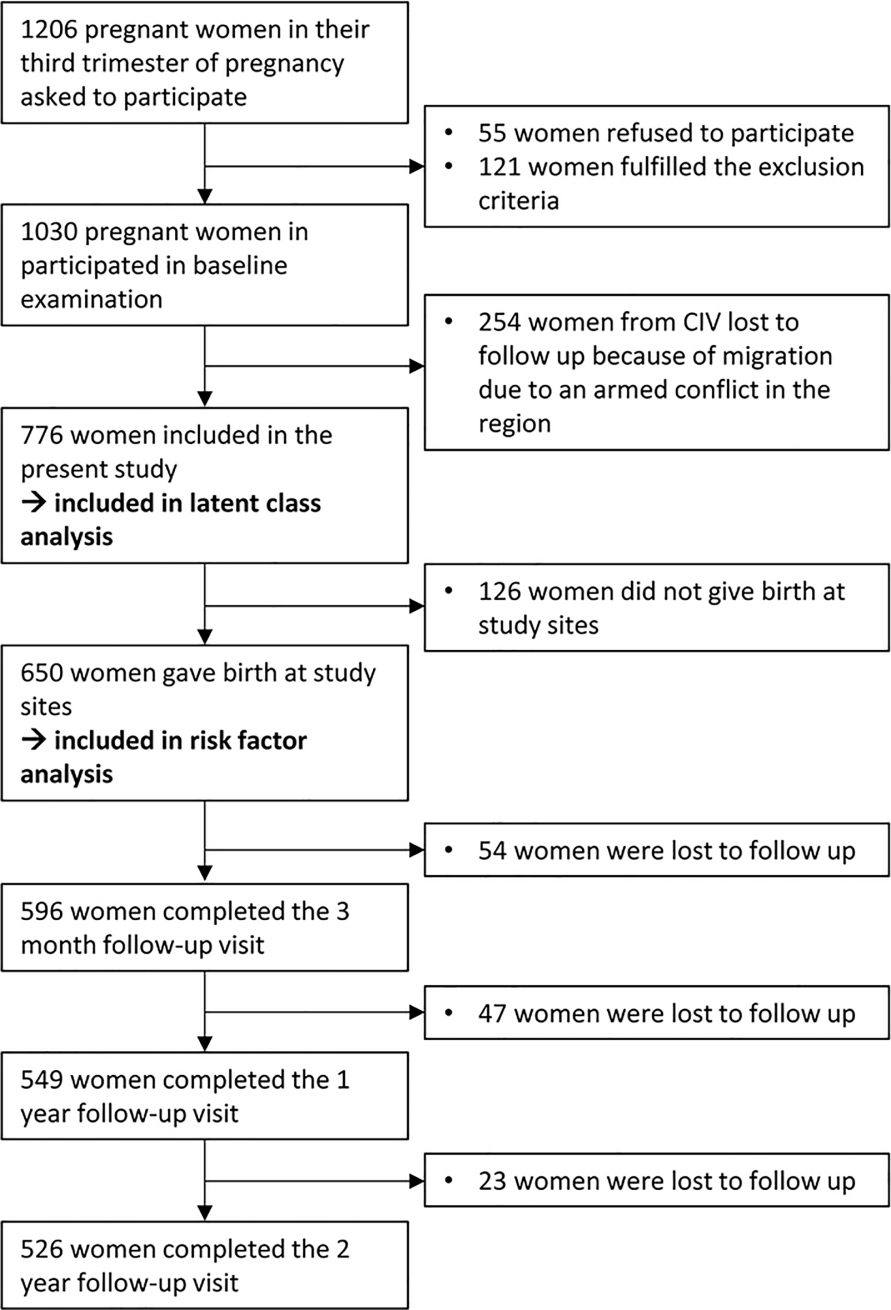
Recruitment flow chart.

Pregnant women had to fulfill the following criteria for inclusion: residing within 5 km around the hospitals, willingness and ability to participate in follow-up measurements, and providing written informed consent. Women were not eligible to participate in case of multiple pregnancy, severe pregnancy complications (e.g., hypertension, hemorrhage, pre-eclampsia, and diabetes), or age under 18 years. Mother-child dyads were excluded when the children met one of the following exclusion criteria at birth: severe perinatal malformation or life-threatening condition, an Apgar score less than 5 after 1 minute and less than 7 after 5 minutes, intrauterine or early postpartum death, premature birth under 34 gestational weeks, a birth weight less than 2.5 kg, or massive clinical jaundice. The recruitment flow chart of the study is presented in Fig 1.

### Variables and instruments

The Patient Health Questionnaire 9 (PHQ-9) was used for the assessment of depressive symptoms 3 months before and 3, 12, and 24 months after delivery [31]. The items rate the frequency of the symptoms of major depressive disorder according to the Diagnostic and Statistical Manual of Mental Disorders [32] within the last two weeks. The questionnaire uses a four-point response scale (0 = *not at all*, 1 = *several days*, 2 = *more than half the days*, and 3 = *nearly every day*), with the sum score ranging from 0 to 27. We previously demonstrated adequate psychometric properties of the PHQ-9 in our study population, with a Cronbach’s α of .65 for the Ivorian sample and a Cronbach’s α of .68 in the Ghanaian sample [33].

The seven-item Generalized Anxiety Disorder scale (GAD-7) was utilized for the assessment of anxiety symptoms 3 months before and 3, 12, and 24 months after delivery [34]. The GAD-7 uses the same response format and recall period as the PHQ-9 and its sum score ranges from 0 to 21. We found the psychometric properties of the GAD-7 adequate in our study population [35]. Measures of internal consistency were Cronbach’s α = .69 in CIV and .67 in Ghana [35].

To assess the level of perceived disability in the last month, we used the 12-item version of the WHO-DAS II [36]. Items are answered on a five-point scale.

Other measurements were administered retrospectively 24 months after birth covering the time period since the birth of the child, including nine items with a dichotomous response format from Ramchandani and colleagues [16]. This measure assesses economic stress (having too little money, being in serious debt, having to support family in financial need), marital stress (relationship breakdown or partner violence), family stress (being seriously ill or having a family member who is, dead of a beloved person), and experience of violence (in danger of being killed or witness of a violent crime). To capture perceived social support, women answered three items on the quality of social support, each by the woman’s mother and by the partner on a three-point scale [17, 37]. Health care utilization was operationalized in our study by the number of sick child visits during the 2 years of study duration in our study facility. This service was free of charge and provided by the CDS study team for the participating mothers for the duration of the study.

Gestational age, Apgar score, sex, and weight of the child were assessed at birth. At study inclusion (3 months prior to delivery), women were asked to provide information on the number of previous pregnancies, complications during previous pregnancies, and previous Caesarian sections. Maternal age, the number of children from relatives or acquaintances living in the same household, and the socioeconomic status (SES) were also assessed at recruitment. We described the SES index in detail elsewhere [38].

### Statistical analysis

We used growth mixture modeling (GMM) to identify latent trajectories of maternal depressive symptoms from 3 months before to 24 months after delivery. Women were included in the analysis if they provided data at recruitment 3 months before birth. GMM classifies individuals into distinct groups, so that individuals within groups show more similar developmental trajectories than individuals in different groups [39]. We modelled a quadrilinear course with four measurement points (3 months before and 3, 12, and 24 months after delivery). Several model fit statistics and criteria were used for identifying the best model structure, including Akaike's information criterion (AIC), Bayesian information criterion (BIC) (lower values indicating better fit), Lo-Mendell-Rubin adjusted likelihood ratio test, bootstrap likelihood ratio test (BLRT) (significant values indicate superiority over a solution with one class less), and entropy (values above .7 preferred). GMM analysis was performed with robust full-information maximum likelihood estimation in Mplus version 7.2 (Muthén & Muthén, Los Angeles, CA).

We performed a multinomial logistic regression analysis in a subsample of women who completed at least two assessments: the first assessment (recruitment prior to birth) and the second assessment (giving birth in one of the study sites) to examine which factors were associated with class membership. Tested variables included psychosocial, obstetric, and sociodemographic factors.

Since a non-negligible amount of data (explanatory variables) were missing, we used multiple imputation methods to complete the dataset. Fifty imputed datasets were generated on the basis of all available information from the modeled and several auxiliary variables. The regression analysis was performed in the datasets separately, and the results were combined using Rubin’s rules [40]. We performed a sensitivity analysis without imputation to test the robustness of our findings. Imputation and regression were performed using SPSS 20.0 [41].

In order to examine longitudinal associations between depressive and anxiety symptoms, the obtained class membership regarding the course of depressive symptoms was cross-tabulated with class membership regarding the course of anxiety symptoms as reported previously [38].

## Results

### Sample

776 women provided sufficient data for inclusion in the analyses, of whom 488 came from CIV and 288 from GHA. Characteristics of the sample are depicted in Table 1. Women from CIV were largely comparable to women from GHA but had less education and were Muslims more frequently.

**Table 1:** Sample characteristics.

Variable	Total (*N* = 776)	CIV (*n* = 488)	GHA (*n* = 288)
Age in years			
* M* (*SD*; range)	28.9 (5.5; 18 to 46)	28.3 (5.8; 18 to 46)	29.8 (4.8; 18 to 42)
Gestational age at birth in weeks			
* M (SD;* range)	39.7 (1.5; 35 to 43)	39.7 (1.6; 35 to 43)	39.7 (1.3; 36 to 43)
Education^1^ *n* (%)			
None	212 (27.4)	200 (41.0)	12 (4.2)
Basic	235 (30.3)	136 (27.9)	99 (34.5)
Secondary	198 (25.5)	119 (24.4)	79 (27.5)
Tertiary	130 (16.8)	33 (6.8)	97 (33.8)
Religion *n* (%)			
Christian	520 (67.0)	249 (51.0)	271 (94.1)
Muslim	244 (31.4)	227 (46.5)	17 (5.9)
None	5 (0.6)	5 (1.0)	0 (0.0)
Traditional African^2^	4 (0.5)	4 (0.8)	0 (0.0)
Other	3 (0.4)	3 (0.6)	0 (0.0)
Marital status *n* (%)			
Currently married	540 (69.6)	314 (64.3)	226 (78.5)
Cohabiting	153 (19.7)	104 (21.3)	49 (17.0)
Never married	58 (7.5)	48 (9.8)	10 (3.5)
Separated	22 (2.8)	21 (4.3)	1 (0.3)
Widowed	2 (0.3)	1 (0.2)	1 (0.3)
Divorced	1 (0.1)	0 (0.0)	1 (0.3)
Number of pregnancy *n* (%)			
First	175 (24.2)	98 (22.2)	77 (27.4)
Second	157 (21.7)	90 (20.3)	67 (23.8)
Third	144 (19.9)	96 (21.7)	48 (17.1)
Fourth	94 (13.0)	56 (12.6)	38 (13.5)
Fifth or more	154 (21.2)	103 (23.2)	51 (18.2)
Previous Caesarian sections *n* (%)			
No	646 (88.7)	429 (94.1)	217 (79.8)
One	65 (8.9)	23 (5.0)	42 (15.4)
Two	15 (2.1)	4 (0.8)	11 (0.7)
Three	2 (0.3)	0 (0.0)	2 (0.7)

*Note*. CIV = Côte d’Ivoire; GHA = Ghana; *M* = mean; *SD* = standard deviation; different *n* due to missing data.

^1^ Primary education refers to the attendance of an elementary school where children acquire basic education. Secondary education describes the attendance of middle schools and/or high schools. Tertiary education refers to higher education, for instance training at a university.

^2^ Traditional African religion is an umbrella term for different spiritual or traditional indigenous religions.

### Distinct trajectory classes

Considering the above mentioned fit statistics and the resulting size of the classes, the three class solution was the best fitting model (Table 2). While entropy was higher for the model with two classes, the BIC and the adjusted likelihood ratio test supported the model with three classes. Although the AIC decreased further for models with more classes, in terms of parsimony, we decided to retain the three class model.

**Table 2:** Fit indices used to identify number of latent classes.

Latent classes	AIC	BIC	Entropy	BLRT	Lo-Mendell-Rubin adjusted LRT	*n* (%) of smallest class
1	13652.754	13689.988				
2	13560.821	13621.325	0.844	<0.001	0.005	37 (4.77)
3	13476.733	13560.508	0.812	<0.001	0.018	33 (4.25)
4	13455.152	13562.197	0.756	<0.001	0.181	29 (3.74)
5	13424.835	13555.151	0.766	<0.001	0.073	16 (2.06)
6*	13401.426	13555.013	0.719	<0.001	0.740	4 (0.52)

*Note*. AIC = Akaike information criterion; BIC = Bayesian information criterion; BLRT = parametric

bootstrapped likelihood ratio test; LRT = Lo-Mendell-Rubin adjusted likelihood ratio test; *due to local maxima no stable class structure was found.

**Fig 2:**
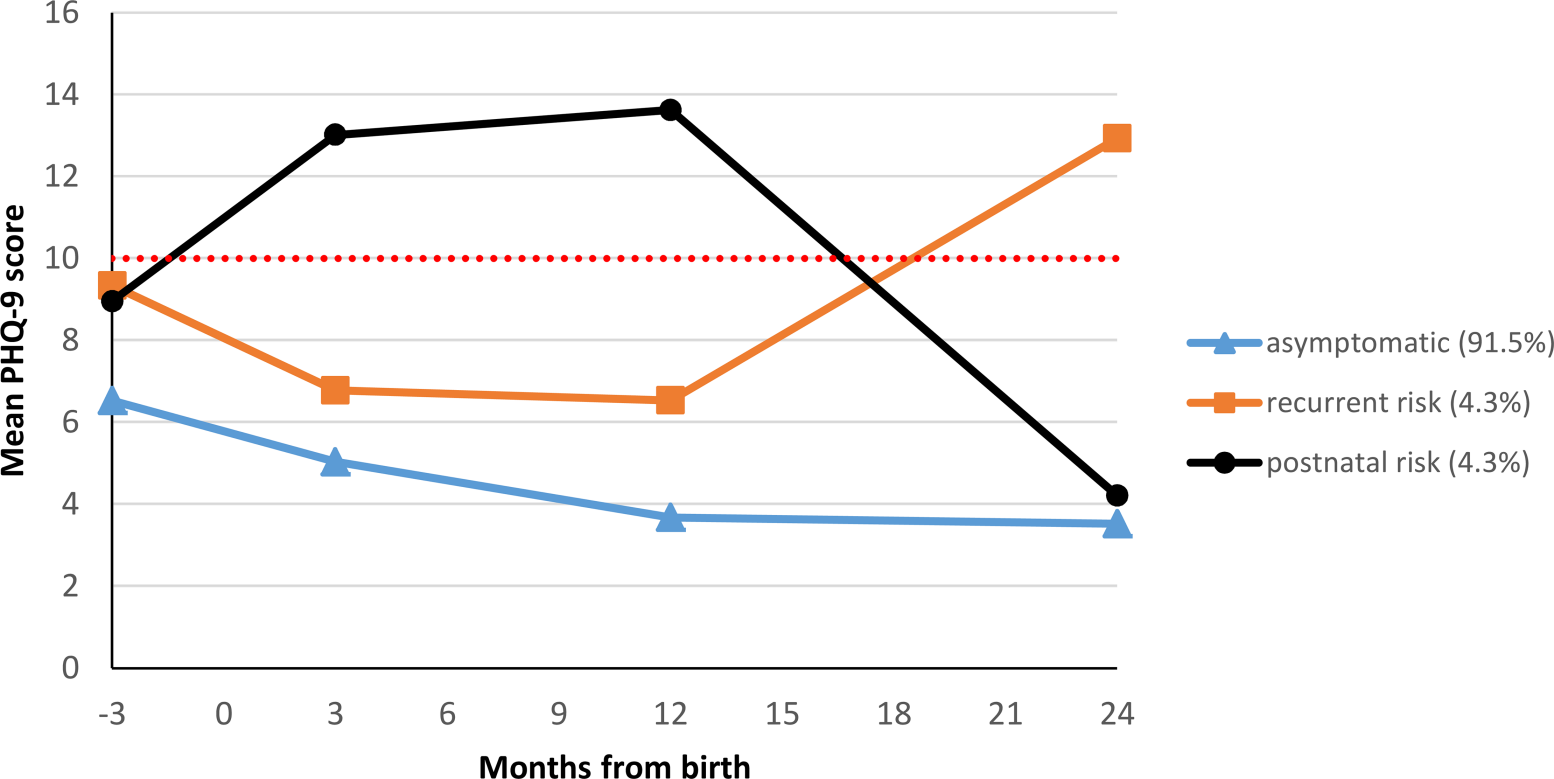
Three classes of depressive symptoms from 3 months before to 24 months after delivery. The dotted line represents the cut-off point of the PHQ-9, above which symptom severity can be considered clinically relevant [31].

The country of origin did not influence maternal depressive symptoms trajectories. We identified identical classes in CIV and GHA. Fig 2 shows the three identified classes of depressive symptom trajectories.

The *asymptomatic* class (class 1) was the largest and comprised 91.5% of women (*n* = 710) who had stable low depressive symptoms during the last trimester of pregnancy and the 2 years following childbirth. The estimated mean PHQ-9 score of these women was 5.7 points at birth and remained low with a slightly decreasing tendency over time (3.5 PHQ-9 points 2 years after birth). The *recurrent risk* class (class 2) comprised 4.3% of women (*n* = 33). They had an estimated average PHQ-9 score of 7.8 points at birth. Their depressive symptomatology decreased slightly and reached a score of 6.5 one year after birth. After that, the PHQ-9 score increased gradually up to 12.9 points 2 years after birth. The *postnatal risk* class (class 3) included 4.3% of women (*n* = 33) with an estimated mean of 11.4 PHQ-9 points at birth. PHQ-9 scores increased with a peak of 13.6 one year after birth. Afterwards, the depressive symptoms decreased and were at an asymptomatic level of 4.2 PHQ-9 points 2 years after birth. Both risk classes were marginally more frequently observed in CIV than in GHA (see S1 Table).

### Associations with trajectory class membership

A subsample of *N* = 650 women who completed the inclusion assessment and gave birth to their children in the study hospitals, was used for the multivariate multinomial logistic regression analysis assessing factors associated with class membership. S2 Table shows descriptive information for all variables investigated in the regression analysis. Collinearity analysis indicated satisfactorily independence of the explanatory variables with pairwise correlations below *r* = .57, tolerance values above .56, and variance inflation factors below 1.79. Results are shown in Table 3. The *asymptomatic* class was used as reference group.

**Table 3:** Association of the investigated factors with class membership (*n* = 650).

Variables	Recurrent risk (class 2)		Postnatal risk (class 3)	
	OR (95% CI)	p	OR (95% CI)	p
Psychosocial factors				
Anxiety symptoms^1^^,^ ^a^	1.13 (1.03, 1.25)	**.014**	1.12 (1.01, 1.24)	**.028**
Disability^1^^,^ ^b^	1.02 (0.96, 1.09)	.525	1.06 (1.00, 1.12)	.063
Partner support^3^^,^ ^c^	0.81 (0.64, 1.03)	.081	0.94 (0.72, 1.21)	.613
Mother support^3^^,^ ^c^	1.04 (0.84, 1.29)	.740	1.01 (0.81, 1.25)	.953
Economic stress^3^^,^ ^d^	1.93 (1.25, 2.96)	**.003**	1.22 (0.79, 1.88)	.371
Marital stress^3^^,^ ^e^	0.60 (0.24, 1.51)	.277	0.71 (0.27, 1.88)	.490
Family stress^3^^,^ ^e^	1.31 (0.74, 2.30)	.355	1.98 (1.06, 3.68)	**.031**
Experience of violence^3^^,^ ^e^	1.05 (0.45, 2.46)	.916	0.69 (0.23, 2.13)	.523
Sociodemographic factors				
Country^1^^,^ ^f^	0.38 (0.12, 1.21)	.101	0.14 (0.03, 0.56)	**.006**
Age of woman in years^1^	1.07 (0.97, 1.17)	.165	1.01 (0.92, 1.10)	.881
SES^1^^,^ ^g^	1.21 (0.40, 3.68)	.735	1.53 (0.51, 4.64)	.450
Educational experience^1^^,^ ^h^	0.61 (0.22, 1.66)	.330	0.35 (0.12, 1.05)	.061
Marital status^1^^,^ ^j^	1.00 (0.26, 3.92)	.99	0.90 (0.26, 3.17)	.872
Number of children from relatives or acquaintances living in household^3^^,^ ^k^	1.00 (0.68, 1.49)	.990	0.90 (0.63, 1.30)	.578
Obstetric factors				
Gestational age in weeks^2^	0.91 (0.67, 1.23)	.541	0.77 (056, 1.05)	.093
Apgar score after 1 minute^2^^,^ ^m^	1.31 (0.49, 3.50)	.594	0.86 (0.34, 2.18)	.757
Sex of child^2^^,^ ^n^	1.35 (0.58, 3.15)	.486	0.86 (0.37, 1.99)	.723
Weight of child in kg^2^	1.32 (0.52, 3.34)	.553	0.34 (0.11, 1.07)	.066
Caesarian section current birth^2^^,^ ^p^	2.12 (0.67, 6.67)	.210	1.10 (0.24, 5.18)	.900
Number of pregnancies before inclusion^1^	1.04 (0.82, 1.33)	.744	1.06 (0.83, 1.37)	.631
Pregnancy complications before inclusion^1^^,^ ^p^	0.44 (0.14, 1.42)	.169	0.98 (0.33, 2.87)	.968
Caesarian section before inclusion^1^^,^ ^p^	0.66 (0.11, 3.94)	.650	0.66 (0.09, 4.61)	.674
Mother pregnant since last birth^3^^,^ ^p^	3.11 (1.10, 8.79)	**.033**	0.55 (0.14, 2.14)	.388
Health care utilization^3^^,^ ^q^	1.04 (0.92, 1.17)	.543	1.09 (0.97, 1.24)	.159

*Note*. The asymptomatic class was used as reference class.

^1^ assessed at inclusion.

^2^ assessed at birth.

^3^ assessed 2 years after birth.

*OR* = odds ratio (*OR* above 1 indicates higher odds of belonging to the respective class compared to the reference class, *OR* below 1 indicates lower odds of belonging to the respective class compared to the reference class); CI = confidence interval.

^a^ GAD-7 sum score: range from 0 to 21; higher scores represent higher level of anxiety symptoms.

^b^ WHO-DAS II sum score: range from 12 to 60; higher scores represent higher level of disability.

^c^ Range from 0 to 6; 3 items answered on a three point scale; higher scores represent higher level of support.

^d^ Range from 0 to 3; 3 items with a dichotomous response format (0 = no; 1 = yes); higher scores represent higher level of stress.

^e^ Range from 0 to 2; 2 items with a dichotomous response format (0 = no; 1 = yes); higher scores represent higher level of stress.

^f^ 1 = CIV; 2 = GHA.

^g^ SES: The median was used to differentiate between low (0) and high (1).

^h^ dichotomized: 0 = none formal education and primary education; 1 = secondary education and tertiary education

^j^ dichotomized: 0 = never married, separated, divorced, or widowed; 1 = currently married or cohabiting

^k^ Wording of item: “Are there any other children of relatives/acquaintances in your household that you have to take care of?”

^m^ Apgar score after 1 minute: dichotomized: 0 = 5 to 8; 1 = 9 to 10.

^n^ 1 = male; 2 = female.

^p^ 0 = no; 1 = yes.

^q^ Number of unscheduled visits of women with women’s sick children.

The risk of belonging to the *recurrent risk* class was increased when women reported high anxiety symptoms 3 months prior to birth and high levels of economic stress. Furthermore, women who belonged to this class were more likely to become pregnant again 2 years after the birth of the child. None of the investigated factors showed protective influences on class membership.

Women with higher anxiety scores during the third trimester of pregnancy and with high levels of family stress had a higher risk of belonging to the *postnatal risk* class. Weight of the child (*p* = .066) and educational experience (*p* = .061) were two factors closed to being significantly associated with membership of the *postnatal risk* class. Ghanaian women were less frequently found in this class compared to the *asymptomatic* class.

The sensitivity analysis (S3 Table) without imputed data largely confirmed the primary findings. All point estimates were within the 95% confidence interval with the exception of the factors health care utilization (for the *recurrent risk* class), mother support and marital status (both for the *postnatal risk* class).

### Relationship of longitudinal depressive symptoms with longitudinal anxiety symptoms

We contrasted the results with findings on the longitudinal trajectories of anxiety symptoms in the same sample of West African women [38]. In this prior study, we employed GMM as well and identified four classes of anxiety symptom trajectories, namely stable low, increasing, decreasing, and transient anxiety symptoms [38]. Contrasting findings on the course of depressive symptoms with the results on the course of anxiety symptoms showed a statistically significant and strong association between the longitudinal courses of depressive and anxiety symptoms (*χ*^2^ = 258.54, *df* = 6, *p*<0.001; *φ* = .577). As depicted in Table 4, the vast majority of 94.5% of the women who belonged to the depressive *asymptomatic* class were either in the stable low or the decreasing anxiety class. Approximately one half (48.5%) of the women with a *recurrent risk* of depression also showed increasing anxiety symptoms, while the other half (51.5%) belonged either to the stable low or the decreasing anxiety class. Finally, 36.4% of the women with a *postnatal risk* for depression were classified as also presenting transient anxiety symptoms, with 39.4% exhibiting stable low, 21.2% showing decreasing, and 3.0% presenting increasing anxiety.

**Table 4:** Relationship of trajectorie0073 of depressive symptoms with trajectories of anxiety symptoms.

		Trajectories of anxiety symptoms			
		Stable low *n* (%)	Increasing *n* (%)	Decreasing *n* (%)	Transient *n* (%)
**Trajectories of depressive symptoms**	**Asymptomatic** *n* (%)	597 (84.1%)	25 (3.5%)	74 (10.4%)	14 (2.0%)
	**Recurrent risk** *n* (%)	9 (27.3%)	16 (48.5%)	8 (24.2%)	0 (0.0%)
	**Postnatal risk** *n* (%)	13 (39.4%)	1 (3.0%)	7 (21.2%)	12 (36.4%)

*Note*. The four classes of longitudinal trajectories of anxiety symptoms stem from the Child Development Study [38].

## Discussion

### Summary of findings and their relation to existing evidence

This is the first study investigating the longitudinal course of perinatal depressive symptoms in two lower income countries in West Africa.

In accordance with previous findings [14, 15, 42], we found the largest group to be an *asymptomatic* group that is characterized by stable low depressive symptoms in the time period from 3 months before until 24 months after birth. However, almost every tenth woman belonged to a risk group. We found two distinct risk groups in our sample, namely the *recurrent risk* class and the *postnatal risk* class.

Even though women from the *recurrent risk* class showed slightly higher depression scores than women from the *asymptomatic* class throughout the first 12 months after birth, their burden was clearly elevated 3 months prior to birth and reached clinical significance 24 months after birth. At that time-point, women of this group were pregnant again more often. We believe that the state of pregnancy per se and a short inter-pregnancy interval may have contributed to their symptoms. In sub-Saharan Africa, pregnancy clearly constitutes a threat to maternal health and well-being. In addition, birth spacing of less than 36 months has been found to contribute to pregnancy risks, adverse birth outcome and socioeconomic inequality [43]. All these factors may have promoted depressive mood during pregnancies in a subgroup of women. Indeed, high levels of economic stress were associated with a *recurrent risk* of depressive symptoms.

Women from the *postnatal risk* class were characterized by a high level of depressive symptoms throughout the first postnatal year with decreased symptoms 24 months after delivery. They exhibited high levels of family stress more often. Living in CIV was also associated with membership in the *postnatal risk* class compared with the asymptomatic class. These findings`characteristics point to “classical” prolonged postpartum depressiveness and may be explained by phase-specific unease in the transition to parenthood, lack of self-confidence while caring for a (new) infant, and challenging changes within family relationships.

Interestingly, women from both classes could not be differentiated from each other based on their PHQ-9 score 3 months prior to birth but only by its postnatal course and based on their specific risk factors. Our results, in line with others [14, 15, 42], suggest that the perinatal phase is quite dynamic in terms of depressive symptomatology since we observed increase (*recurrent risk* class) and decrease (*postnatal risk* class) of depressive symptoms throughout the second year after birth.

In accordance with previous findings [12–14], higher levels of anxiety symptoms were associated with the membership of both risk classes in our sample. Comorbidity between depression and anxiety has been documented in ante- and postpartum women [44, 45]. In particular, depressive and anxiety symptoms were also longitudinally associated in our sample. The majority of women had clinically irrelevant symptom levels in both disease groups. However, approximately half of the women from the *recurrent risk* group for depressive symptoms also showed higher longitudinal levels of anxiety symptoms. This finding highlights the double burden of impaired mood for those affected. More than one third of women from our *postnatal risk* depressive symptoms class belonged to the *asymptomatic* anxiety symptoms class. However, one third had transient symptoms of both disorders.

We found, like some others, tendentious associations of depressive symptoms with maternal educational experience [20] and weight of the child although prematurity and low birth weight had led to study exclusion. We did not find any associations of belonging to a depressive risk class with maternal age [17, 26] and parity [20, 23, 25].

In contrast to prior findings, we did not see an association between perinatal depressive symptoms and marital stress (prevalence 17.6%) [14, 19]; and we also did not find and association between perinatal depressive symptoms and the experience of violence (prevalence 12.0%) [16, 18, 19]. Furthermore, there were no associations between the level of social support either provided by the mother or partner of the women and perinatal depressive symptoms. We did also not detect associations of class membership with marital status and the experience of complications during previous pregnancies [20, 21, 26, 27].

### Limitations

Our study has limitations. First, some constructs such as perceived social support and stress were assessed retrospectively 2 years after the birth of the child. The assessment of these constructs might be prone to recall bias. Second, some factors such as health care utilization and new pregnancy could rather be an effect of the class membership instead of a cause. Third, the validity of assessing health care utilization might be limited because we could only count sick child visits in both designated study clinics, available free of charge for study participants. No data were available regarding cases where mothers sought help from other health care providers. Fourth, we were not able to differentiate between women who suffered from depression before pregnancy and women who developed depressive symptoms during the course of the study [14]. Fifth, depression and anxiety were not assessed by clinical diagnostic interviews, but by screening instruments. However, the PHQ-9 and the GAD-7 are widely accepted and the validity of the instruments has been established in our study population [33, 35].

Sixth, it is unclear to which degree our sample represents pregnant women in rural West Africa and without access to healthcare, since we investigated women from two urban regions of CIV and Ghana. According to the World Bank Report [46], approximately half of the population in both countries live in rural regions (urban population of 54.87% in CIV and 54.68% in Ghana, both in 2016). Presumably, women from rural areas are at least in part exposed to other stressors and risk factors (e.g., limited access to health care with less well-trained personnel, lower educational attainment), but also to potentially protective factors available (e.g., social support provided by members of larger family structures).

Seventh, the inclusion criteria may have led to the exclusion of some women at potentially high risk of developing depressive symptoms. Women with children under 2.5 kg birthweight and babies born before the 34^th^ week of gestation could not participate in our study. According to the Demographic and Health Survey, 14.2% of Ivorian children [47] and 10% of Ghanaian children [48] are born with less than 2.5 kg birthweight. Low birthweight and low gestational age can be risk factors for postnatal depression [49, 50]. Thus, we may have underestimated the prevalence of depressive symptoms in West African women.

Eighth, we did not adjust for the inflation of the type I error due to multiple testing. This would have led to a considerable increase in type II error frequency, which would have been incompatible with the explorative character of the study. Because of limited power of the risk factor analyses due to the small number of women at risk in the sample, we consider this analytic strategy adequate for balancing tradeoffs between type I and type II errors.

### Conclusions

Our data indicate that a substantial proportion of West African women developed an unfavorable course of depressive symptoms during the vulnerable phase of pregnancy and early motherhood. Different patterns of symptom occurrence can be distinguished and categorized clinically. Similar to mothers in high income countries, psychosocial factors appear to play a decisive role. Particularly, the strong association with anxiety symptoms calls for attention of policy makers in West African countries and beyond. In addition, even in an economically impoverished environment, perceived economic hardship further increased the mental health burden in our study population.

## Supporting information

S1 Table Class membership for total sample and both study sites separately. (DOCX)

S2 Table Sample characteristics regarding predictor variables used in the multinomial logistic regression analysis. (DOCX)

S3 Table Sensitivity analysis using cases with completed data on all variables (*n* = 650). (DOCX)

## References

[1] Walker SP, Wachs TD, Gardner JM, Lozoff B, Wasserman GA, Pollitt E, et al Child development: risk factors for adverse outcomes in developing countries. Lancet. 2007;369(9556):145–57. doi: 10.1016/S0140-6736(07)60076-2 17223478 10.1016/S0140-6736(07)60076-2

[2] Prince M, Patel V, Saxena S, Maj M, Maselko J, Phillips MR, et al No health without mental health. Lancet. 2007;370(9590):859–77. doi: 10.1016/S0140-6736(07)61238-0 17804063 10.1016/S0140-6736(07)61238-0

[3] Sawyer A, Ayers S, Smith H. Pre-and postnatal psychological wellbeing in Africa: a systematic review. J Affect Disord. 2010;123(1):17–29. 19635636 10.1016/j.jad.2009.06.027

[4] Goodman SH, Rouse MH, Connell AM, Broth MR, Hall CM, Heyward D. Maternal depression and child psychopathology: A meta-analytic review. Clin Child Fam Psychol Rev. 2011;14(1):1–27. doi: 10.1007/s10567-010-0080-1 21052833 10.1007/s10567-010-0080-1

[5] Guo N, Bindt C, Te Bonle M, Appiah-Poku J, Hinz R, Barthel D, et al Association of antepartum and postpartum depression in Ghanaian and Ivorian women with febrile illness in their offspring: a prospective birth cohort study. Am J Epidemiol. 2013;178:1394–402. doi: 10.1093/aje/kwt142 24013202 10.1093/aje/kwt142

[6] Barry TJ, Murray L, Fearon RP, Moutsiana C, Cooper P, Goodyer IM, et al Maternal postnatal depression predicts altered offspring biological stress reactivity in adulthood. Psychoneuroendocrinology. 2015;52:251–60. doi: 10.1016/j.psyneuen.2014.12.003 25544737 10.1016/j.psyneuen.2014.12.003 PMC4309884

[7] Black MM, Walker SP, Fernald LC, Andersen CT, DiGirolamo AM, Lu C, et al Early childhood development coming of age: science through the life course. Lancet. 2016. 10.1016/S0140-6736(16)31389-7 PMC5884058 27717614

[8] World Health Organization. Mental health included in the UN Sustainable Development Goals 2016 [2016, November 30]. Available from: http://www.who.int/mental_health/SDGs/en/.

[9] Herba CM, Glover V, Ramchandani PG, Rondon MB. Maternal depression and mental health in early childhood: an examination of underlying mechanisms in low-income and middle-income countries. Lancet Psychiatry. 2016;3(10):983–92. doi: 10.1016/S2215-0366(16)30148-1 27650772 10.1016/S2215-0366(16)30148-1

[10] World Health Organization. The global burden of disease: 2004 update. Geneva: World Health Organization; 2008.

[11] Patel V, Kim Y-R. Contribution of low-and middle-income countries to research published in leading general psychiatry journals, 2002–2004. BJPsych. 2007;190(1):77–8. 17197661 10.1192/bjp.bp.106.025692

[12] Heron J, O'Connor TG, Evans J, Golding J, Glover V, Team AS. The course of anxiety and depression through pregnancy and the postpartum in a community sample. J Affect Disord. 2004;80(1):65–73. doi: 10.1016/j.jad.2003.08.004 15094259 10.1016/j.jad.2003.08.004

[13] Lee AM, Lam SK, Lau SMSM, Chong CSY, Chui HW, Fong DYT. Prevalence, course, and risk factors for antenatal anxiety and depression. Obstetrics & Gynecology. 2007;110(5):1102–12. 17978126 10.1097/01.AOG.0000287065.59491.70

[14] Martini J, Petzoldt J, Einsle F, Beesdo-Baum K, Höfler M, Wittchen H-U. Risk factors and course patterns of anxiety and depressive disorders during pregnancy and after delivery: A prospective-longitudinal study. J Affect Disord. 2015;175:385–95. doi: 10.1016/j.jad.2015.01.012 25678171 10.1016/j.jad.2015.01.012

[15] Mora PA, Bennett IM, Elo IT, Mathew L, Coyne JC, Culhane JF. Distinct trajectories of perinatal depressive symptomatology: evidence from growth mixture modeling. Am J Epidemiol. 2009;169(1):24–32. doi: 10.1093/aje/kwn283 19001135 10.1093/aje/kwn283 PMC2720701

[16] Ramchandani PG, Richter LM, Stein A, Norris SA. Predictors of postnatal depression in an urban South African cohort. J Affect Disord. 2009;113(3):279–84. doi: 10.1016/j.jad.2008.05.007 18571734 10.1016/j.jad.2008.05.007

[17] Hartley M, Tomlinson M, Greco E, Comulada WS, Stewart J, Le Roux I, et al Depressed mood in pregnancy: prevalence and correlates in two Cape Town peri-urban settlements. Reprod Health. 2011;8(9):4755–8. 10.1186/1742-4755-8-9 PMC3113332 21535876

[18] Tsai AC, Tomlinson M, Comulada WS, Rotheram-Borus MJ. Intimate partner violence and depression symptom severity among South African women during pregnancy and postpartum: population-based prospective cohort study. PLoS Med. 2016;13(1):e1001943 doi: 10.1371/journal.pmed.1001943 26784110 10.1371/journal.pmed.1001943 PMC4718639

[19] Nasreen HE, Kabir ZN, Forsell Y, Edhborg M. Prevalence and associated factors of depressive and anxiety symptoms during pregnancy: a population based study in rural Bangladesh. BMC Womens Health. 2011;11(1):1. 21635722 10.1186/1472-6874-11-22 PMC3117808

[20] Ali NS, Azam IS, Ali BS, Tabbusum G, Moin SS. Frequency and associated factors for anxiety and depression in pregnant women: a hospital-based cross-sectional study. The Scientific World Journal. 2012. 10.1100/2012/653098 PMC3354685 22629180

[21] Fisher J, Tran T, Tran TD, Dwyer T, Nguyen T, Casey GJ, et al Prevalence and risk factors for symptoms of common mental disorders in early and late pregnancy in Vietnamese women: a prospective population-based study. J Affect Disord. 2013;146(2):213–9. doi: 10.1016/j.jad.2012.09.007 23026129 10.1016/j.jad.2012.09.007

[22] Abuidhail J, Abujilban S. Characteristics of Jordanian depressed pregnant women: a comparison study. J Psychiatr Ment Health Nurs. 2014;21(7):573–9. doi: 10.1111/jpm.12125 24299168 10.1111/jpm.12125

[23] Golbasi Z, Kelleci M, Kisacik G, Cetin A. Prevalence and correlates of depression in pregnancy among Turkish women. Matern Child Health J. 2010;14(4):485–91. doi: 10.1007/s10995-009-0459-0 19238527 10.1007/s10995-009-0459-0

[24] Husain N, Parveen A, Husain M, Saeed Q, Jafri F, Rahman R, et al Prevalence and psychosocial correlates of perinatal depression: a cohort study from urban Pakistan. Arch Womens Ment Health. 2011;14(5):395–403. doi: 10.1007/s00737-011-0233-3 21898171 10.1007/s00737-011-0233-3

[25] Dibaba Y, Fantahun M, Hindin MJ. The association of unwanted pregnancy and social support with depressive symptoms in pregnancy: evidence from rural Southwestern Ethiopia. BMC Pregnancy Childbirth. 2013;13(1):1. 23800160 10.1186/1471-2393-13-135 PMC3716614

[26] Weobong B, Soremekun S, ten Asbroek AH, Amenga-Etego S, Danso S, Owusu-Agyei S, et al Prevalence and determinants of antenatal depression among pregnant women in a predominantly rural population in Ghana: The DON population-based study. J Affect Disord. 2014;165:1–7. doi: 10.1016/j.jad.2014.04.009 24882170 10.1016/j.jad.2014.04.009

[27] Brittain K, Myer L, Koen N, Koopowitz S, Donald KA, Barnett W, et al Risk factors for antenatal depression and associations with infant birth outcomes: Results from a South African birth cohort study. Paediatr Perinat Epidemiol. 2015;29(6):505–14. doi: 10.1111/ppe.12216 26236987 10.1111/ppe.12216

[28] Bindt C, Appiah-Poku J, Te Bonle M, Schoppen S, Feldt T, Barkmann C, et al Antepartum depression and anxiety associated with disability in African women: cross-sectional results from the CDS study in Ghana and Côte d'Ivoire. PloS one. 2012;7(10):e48396 doi: 10.1371/journal.pone.0048396 23110236 10.1371/journal.pone.0048396 PMC3482210

[29] Kone-Pefoyo A, Rivard M. Poverty and sociocultural factors in the use of maternal health services in Ivory Coast. Revue d'epidemiologie et de sante publique. 2006;54(6):485–95. 17194980 10.1016/s0398-7620(06)76748-7

[30] Arthur E. Wealth and antenatal care use: implications for maternal health care utilisation in Ghana. Health Economics Review. 2012;2(1):14 doi: 10.1186/2191-1991-2-14 22866869 10.1186/2191-1991-2-14 PMC3484029

[31] Kroenke K, Spitzer RL, Williams JB. The PHQ‐9. J Gen Intern Med. 2001;16(9):606–13. doi: 10.1046/j.1525-1497.2001.016009606.x 11556941 10.1046/j.1525-1497.2001.016009606.x PMC1495268

[32] American Psychiatric Association. Diagnostic and statistical manual of mental disorders. 4th ed. Washington, DC, 1994.

[33] Barthel D, Barkmann C, Ehrhardt S, Schoppen S, Bindt C. Screening for depression in pregnant women from Côte d'Ivoire and Ghana: Psychometric properties of the Patient Health Questionnaire-9. J Affect Disord. 2015;187:232–40. doi: 10.1016/j.jad.2015.06.042 26343851 10.1016/j.jad.2015.06.042

[34] Spitzer RL, Kroenke K, Williams JB, Löwe B. A brief measure for assessing generalized anxiety disorder: the GAD-7. Arch Intern Med. 2006;166(10):1092–7. doi: 10.1001/archinte.166.10.1092 16717171 10.1001/archinte.166.10.1092

[35] Barthel D, Barkmann C, Ehrhardt S, Bindt C, Group ICS. Psychometric properties of the 7-item Generalized Anxiety Disorder scale in antepartum women from Ghana and Côte d’Ivoire. J Affect Disord. 2014;169:203–11. doi: 10.1016/j.jad.2014.08.004 25212996 10.1016/j.jad.2014.08.004

[36] Üstün TB. Measuring health and disability: manual for WHO disability assessment schedule WHODAS 2.0: World Health Organization; 2010.

[37] Cooper PJ, Tomlinson M, Swartz L, Woolgar M, Murray L, Molteno C. Post-partum depression and the mother-infant relationship in a South African peri-urban settlement. BJPsych. 1999;175(6):554–8. 10789353 10.1192/bjp.175.6.554

[38] Barthel D, Kriston L, Barkmann C, Appiah-Poku J, Te Bonle M, Doris KYE, et al Longitudinal course of ante-and postpartum generalized anxiety symptoms and associated factors in West-African women from Ghana and Côte d’Ivoire. J Affect Disord. 2016;197:125–33. doi: 10.1016/j.jad.2016.03.014 26991367 10.1016/j.jad.2016.03.014

[39] Jung T, Wickrama K. An introduction to latent class growth analysis and growth mixture modeling. Soc Personal Psychol Compass. 2008;2(1):302–17.

[40] Rubin DB. Multiple imputation for nonresponse in surveys. Hoboken, NJ: John Wiley & Sons; 2004.

[41] SPSS. IBM SPSS statistics for Windows, version 20.0. IBM Corp. Armonk, NY, 2011.

[42] Sutter-Dallay A, Cosnefroy O, Glatigny-Dallay E, Verdoux H, Rascle N. Evolution of perinatal depressive symptoms from pregnancy to two years postpartum in a low-risk sample: the MATQUID cohort. J Affect Disord. 2012;139(1):23–9. doi: 10.1016/j.jad.2011.08.018 22410506 10.1016/j.jad.2011.08.018

[43] Heaton TB, Crookston B, Pierce H, Amoateng AY. Social inequality and children’s health in Africa: a cross sectional study. Int J Equity Health. 2016;15(1):92. 27301658 10.1186/s12939-016-0372-2 PMC4906977

[44] Field T, Diego M, Hernandez‐Reif M, Schanberg S, Kuhn C, Yando R, et al Pregnancy anxiety and comorbid depression and anger: effects on the fetus and neonate. Depress Anxiety. 2003;17(3):140–51. doi: 10.1002/da.10071 12768648 10.1002/da.10071

[45] Andersson L, Sundström‐Poromaa I, Wulff M, Åström M, Bixo M. Depression and anxiety during pregnancy and six months postpartum: A follow‐up study. Acta Obstet Gynecol Scand. 2006;85(8):937–44. doi: 10.1080/00016340600697652 16862471 10.1080/00016340600697652

[46] World Bank. 2016 [Retrieved August 14th, 2017]. Available from: http://data.worldbank.org/indicator/SP.RUR.TOTL.ZS.

[47] Institut National de la Statistique (INS) et ICF International. Côte d’Ivoire Enquête Démographique et de Santé et à Indicateurs Multiples 2011–2012. Calverton, Maryland, USA: 2012.

[48] Ghana Statistical Service (GSS), Ghana Health Service (GHS), and ICF International. Ghana Demographic and Health Survey 2014. Rockville, Maryland, USA: 2015.

[49] Grote NK, Bridge JA, Gavin AR, Melville JL, Iyengar S, Katon WJ. A meta-analysis of depression during pregnancy and the risk of preterm birth, low birth weight, and intrauterine growth restriction. Arch. Gen. Psychiatry. 2010;67(10):1012–24. doi: 10.1001/archgenpsychiatry.2010.111 20921117 10.1001/archgenpsychiatry.2010.111 PMC3025772

[50] Davalos DB, Yadon CA, Tregellas HC. Untreated prenatal maternal depression and the potential risks to offspring: a review. Arch Womens Ment Health. 2012;15(1):1–14. doi: 10.1007/s00737-011-0251-1 22215285 10.1007/s00737-011-0251-1

