# Supplementary material for: Trajectories of maternal ante- and postpartum depressive symptoms and their association with child- and mother-related characteristics in a West African birth cohort study
Source: PLoS One. 2017 Nov 6;12(11):e0187267. doi: 10.1371/journal.pone.0187267 (PMC5673167; doi:10.1371/journal.pone.0187267)
Supplement: S1 Table — (DOCX) [file pone.0187267.s001.docx]

# Supporting Information

# Trajectories of maternal ante- and postpartum depressive symptoms and their association with child- and mother-related characteristics in a West African birth cohort study

Dana Barthel^1,2*^, Levente Kriston^3^, Daniel Fordjour^4^, Yasmin Mohammed^4^, Kra Yao Esther Doris^5^, Bony Kotchi Carine Esther^6^, Koffi Ekissi Jean Armel^6^, Kirsten Alexandra Eberhardt^1^, Torsten Feldt^1,7^, Rebecca Hinz^1,8^, Mathurin Koffi^9^, Stefanie Schoppen^1^, Carola Bindt^2¶^, Stephan Ehrhardt^1,10¶^, on behalf of the International CDS Study Group^^^

**S1 Table**. Class membership for total sample and both study sites separately

|  | **Total (*N*=776)** | **CIV (*n*=488)** | **GHA (*n*=288)** |
| --- | --- | --- | --- |
| Asymptomatic (class 1) *n* (%) | 710 (91.5) | 436 (89.3) | 274 (95.1) |
| Recurrent risk (class 2) *n* (%) | 33 (4.3) | 23 (4.7) | 10 (3.5) |
| Postnatal risk (class 3) *n* (%) | 33 (4.3) | 29 (5.9) | 4 (1.4) |

*Note*. CIV = Côte d’Ivoire; GHA = Ghana
