# Supplementary material for: Trajectories of maternal ante- and postpartum depressive symptoms and their association with child- and mother-related characteristics in a West African birth cohort study
Source: PLoS One. 2017 Nov 6;12(11):e0187267. doi: 10.1371/journal.pone.0187267 (PMC5673167; doi:10.1371/journal.pone.0187267)
Supplement: S2 Table — (DOCX) [file pone.0187267.s002.docx]

# Supporting Information

# Trajectories of maternal ante- and postpartum depressive symptoms and their association with child- and mother-related characteristics in a West African birth cohort study

Dana Barthel^1,2*^, Levente Kriston^3^, Daniel Fordjour^4^, Yasmin Mohammed^4^, Kra Yao Esther Doris^5^, Bony Kotchi Carine Esther^6^, Koffi Ekissi Jean Armel^6^, Kirsten Alexandra Eberhardt^1^, Torsten Feldt^1,7^, Rebecca Hinz^1,8^, Mathurin Koffi^9^, Stefanie Schoppen^1^, Carola Bindt^2¶^, Stephan Ehrhardt^1,10¶^, on behalf of the International CDS Study Group^^^

**S2 Table.** Sample characteristics regarding predictor variables used in the multinomial logistic regression analysis

| **Variable** | **Total (*N*=650)** | **CIV (*n*=362)** | **GHA (*n*=288)** |
| --- | --- | --- | --- |
| **Psychosocial factors** |  |  |  |
| Anxiety symptoms^1, a^  *M* (*SD*; range) | 5.1 (3.8; 0 to 21) | 5.4 (3.8; 0 to 18) | 4.7 (3.8; 0 to 21) |
| Disability^1, b^  *M* (*SD*; range) | 24.4 (7.0; 12 to 48) | 23.7 (7.1; 12 to 45) | 25.2 (6.8; 12 to 43) |
| Partner support^3, c^  *M* (*SD*; range) | 4.6 (1.8; 0 to 6) | 4.4 (1.9; 0 to 6) | 4.9 (1.8; 0 to 6) |
| Mother support^3, c^  *M* (*SD*; range) | 4.4 (2.1; 0 to 6) | 4.5 (2.0; 0 to 6) | 4.3 (2.2; 0 to 6) |
| Economic stress^3, d^  *M* (*SD*; range) | 0.9 (1.0; 0 to 3) | 0.9 (1.0; 0 to 3) | 0.9 (1.0; 0 to 3) |
| Marital stress^3, e^  *M* (*SD*; range) | 0.2 (0.5; 0 to 2) | 0.3 (0.6; 0 to 2) | 0.1 (0.4; 0 to 2) |
| Family stress^3, e^  *M* (*SD*; range) | 0.8 (0.8; 0 to 2) | 0.9 (0.8; 0 to 2) | 0.8 (0.74; 0 to 2) |
| Experience of violence^3, e^  *M* (*SD*; range) | 0.2 (0.5; 0 to 2) | 0.1 (0.3; 0 to 2) | 0.2 (0.6; 0 to 2) |
| **Sociodemographic factors** |  |  |  |
| Age of woman in years^1^  *M* (*SD*; range) | 29.2 (5.4; 18 to 46) | 28.7 (5.8; 18 to 46) | 29.8 (4.8; 18 to 42) |
| SES^1, f^ *n* (%)  Low  High | 287 (45.0)  351 (55.0) | 248 (69.9)  107 (30.1) | 39 (13.8)  244 (86.2) |
| Educational experience^1, h^ *n* (%)  0  1 | 355 (54.7)  294 (45.3) | 244 (67.4)  118 (32.6) | 111 (38.7)  176 (61.3) |
| Marital status^1, j^ *n* (%)  0  1 | 63 (9.7)  587 (90.3) | 50 (13.8)  312 (86.2) | 13 (4.5)  275 (95.5) |
| Number of children from relatives or acquaintances living in household^3^  *M* (*SD*; range) | 0.6 (1.1; 0 to 7) | 1.0 (1.3; 0 to 7) | 0.08 (0.3; 0 to 2) |
| **Obstetric factors** |  |  |  |
| Gestational age in weeks^2^  *M* (*SD*; range) | 39.7 (1.5; 35 to 43) | 39.7 (1.6; 35 to 43) | 39.7 (1.3; 36 to 43) |
| Apgar score after 1 minute (dichotomized)^2, k^ *n* (%)  Medium  High | 519 (79.8)  131 (20.2) | 237 (65.5)  125 (34.5) | 282 (97.9)  6 (2.1) |
| Sex of child^2^ *n* (%)  Female  Male | 326 (50.2)  324 (49.8) | 166 (45.9)  196 (54.1) | 160 (55.6)  128 (44.4) |
| Weight of child in kg^2^  *M* (*SD*; range) | 3.2 (0.4; 2.5 to 4.9) | 3.1 (0.4; 2.5 to 4.9) | 3.2 (0.4; 2.5 to 4.9) |
| Caesarian section current birth^2^ *n* (%)  Yes  No | 122 (18.8)  526 (80.9) | 26 (7.2)  334 (92.8) | 96 (33.3)  192 (66.7) |
| Number of pregnancies before inclusion^1^ *n* (%)  First  Second  Third  Fourth  Fifth or more | 172 (27.1)  132 (20.8)  116 (18.3)  79 (12.4)  136 (21.4) | 92 (26.4)  63 (18.1)  68 (19.5)  41 (11.7)  85 (24.3) | 80 (28.0)  69 (24.1)  48 (16.8)  38 (13.3)  51 (17.7) |
| Pregnancy complications before inclusion^1^ *n* (%)  Yes  No | 158 (25.2)  470 (74.8) | 65 (18.1)  294 (81.9) | 93 (34.6)  176 (65.4) |
| Caesarian section before inclusion^1^ *n* (%)  Yes  No | 77 (12.6)  536 (87.4) | 22 (6.5)  319 (93.5) | 55 (20.2)  217 (79.8) |
| Mother pregnant since last birth^3^ *n* (%)  Yes  No | 94 (20.1)  374 (79.9) | 44 (16.2)  227 (83.8) | 50 (25.4)  147 (74.6) |
| Health care utilization^3^  *M* (*SD*; range) | 3.6 (3.1; 0 to 16) | 4.0 (3.0; 0 to 15) | 3.2 (3.2; 0 to 16) |

^1^ assessed at inclusion; ^2^ assessed at birth; ^3^ assessed 2 years after birth

^a^ GAD-7 sum score: range from 0 to 21; higher scores represent higher level of anxiety symptoms.

^b^ WHO-DAS II sum score: range from 12 to 60; higher scores represent higher level of disability.

^c^ Range from 0 to 6; 3 items answered on a three point scale; higher scores represent higher level of support.

^d^ Range from 0 to 3; 3 items with a dichotomous response format (0 = no; 1 = yes); higher scores represent higher level of stress.

^e^ Range from 0 to 2; 2 items with a dichotomous response format (0 = no; 1 = yes); higher scores represent higher level of stress.

^f^ SES: The median was used to differentiate between low (0) and high (1).

^h^ dichotomized: 0 = none formal education and primary education; 1 = secondary education and tertiary education

^j^ dichotomized: 0 = never married, separated, divorced, or widowed; 1 = currently married or cohabiting

^k^ Apgar score after 1 minute: dichotomized: 0 = 5 to 8; 1 = 9 to 10.
