# Supplementary material for: Trajectories of maternal ante- and postpartum depressive symptoms and their association with child- and mother-related characteristics in a West African birth cohort study
Source: PLoS One. 2017 Nov 6;12(11):e0187267. doi: 10.1371/journal.pone.0187267 (PMC5673167; doi:10.1371/journal.pone.0187267)
Supplement: S3 Table — (DOCX) [file pone.0187267.s003.docx]

# Supporting Information

# Trajectories of maternal ante- and postpartum depressive symptoms and their association with child- and mother-related characteristics in a West African birth cohort study

Dana Barthel^1,2*^, Levente Kriston^3^, Daniel Fordjour^4^, Yasmin Mohammed^4^, Kra Yao Esther Doris^5^, Bony Kotchi Carine Esther^6^, Koffi Ekissi Jean Armel^6^, Kirsten Alexandra Eberhardt^1^, Torsten Feldt^1,7^, Rebecca Hinz^1,8^, Mathurin Koffi^9^, Stefanie Schoppen^1^, Carola Bindt^2¶^, Stephan Ehrhardt^1,10¶^, on behalf of the International CDS Study Group^^^

**S3 Table**. Sensitivity analysis using cases with completed data on all variables (*n* = 650).

| **Variables** | **Recurrent risk (class 2)** | | **Postnatal risk (class 3)** | |
| --- | --- | --- | --- | --- |
|  | **OR (95% CI)** | **p** | **OR (95% CI)** | **p** |
| Psychosocial factors |  |  |  |  |
| Anxiety symptoms^1, a^ | 1.14 (0.95, 1.36) | .173 | 1.20 (0.98, 1.48) | .075 |
| Disability^1, b^ | 1.02 (0.92, 1.12) | .765 | 0.96 (0.85, 1.09) | .521 |
| Partner support^3, c^ | 0.83 (0.59, 1.16) | .275 | 0.89 (0.52, 1.52) | .663 |
| Mother support^3, c^ | 0.93 (0.71, 1.22) | .609 | 0.74 (0.50, 1.08) | .118 |
| Economic stress^3, d^ | 2.47 (1.32, 4.63) | **.005** | 1.93 (0.92, 4.04) | .080 |
| Marital stress^3, e^ | 0.84 (0.25, 2.84) | .774 | 0.62 (0.10, 4.00) | .613 |
| Family stress^3, e^ | 1.64 (0.75, 3.57) | .215 | 1.14 (0.40, 3.26) | .813 |
| Experience of violence^3, e^ | 1.17 (0.41, 3.37) | .765 | 0.21 (0.02, 2.51) | .219 |
| Sociodemographic factors |  |  |  |  |
| Country^1, f^ | 0.65 (0.12, 3.64) | .621 | n.e. | n.e. |
| Age of woman in years^1^ | 0.96 (0.82, 1.12) | .571 | 1.07 (0.87, 1.32) | .514 |
| SES^1, g^ | 0.55 (0.11, 2.72) | .461 | 1.03 (0.15, 6.95) | .974 |
| Educational experience^1, h^ | 1.32 (0.29, 5.97) | .721 | 0.38 (0.06, 2.63) | .329 |
| Marital status^1, j^ | 0.34 (0.06, 1.95) | .228 | 6.70 (0.44, 102.13) | .171 |
| Number of children from relatives or acquaintances living in household^3, k^ | 0.92 (0.47, 1.79) | .797 | 0.81 (0.42, 1.55) | .520 |
| Clinical-obstetric factors |  |  |  |  |
| Gestational age in weeks^2^ | 0.88 (0.56, 1.39) | .586 | n.e. | n.e. |
| Apgar score after 1 minute^2, l^ | 2.27 (0.44, 11.72) | .327 | 1.32 (0.26, 6.74) | .738 |
| Sex of child^2, m^ | 1.91 (0.47, 7.75) | .367 | 0.90 (0.19, 4.15) | .887 |
| Weight of child in kg^2^ | 4.30 (0.90, 20.63) | .068 | 0.28 (0.03, 2.66) | .268 |
| Caesarian section current birth^2, n^ | 1.30 (0.22, 7.59) | .768 | n.e. | n.e. |
| Number of pregnancies before inclusion^1^ | 1.38 (0.92, 2.08) | .118 | 0.84 (0.45, 1.54) | .568 |
| Pregnancy complications before inclusion^1, n^ | 0.69 (0.12, 3.88) | .674 | 1.00 (0.18, 5.72) | .999 |
| Caesarian section before inclusion^1, n^ | 0.49 (0.03, 7.34) | .603 | n.e. | n.e. |
| Mother pregnant since last birth^3, n^ | 3.40 (0.80, 14.44) | .098 | 0.21 (0.02, 2.46) | .211 |
| Health care utilization^3, p^ | 0.85 (0.66, 1.09) | .192 | 1.18 (0.90, 1.55) | .227 |

*Note*. The asymptomatic class was used as reference class.

^1^ assessed at inclusion; ^2^ assessed at birth; ^3^ assessed 2 years after birth; *OR* = odds ratio (*OR* above 1 indicates higher probability of belonging to the respective class compared to the reference class, *OR* below 1 indicates lower probability of belonging to the respective class compared to the reference class); CI = confidence interval; n.e. = not estimated due to low number of cases.

^a^ GAD-7 sum score: range from 0 to 21; higher scores represent higher level of anxiety symptoms.

^b^ WHO-DAS II sum score: range from 12 to 60; higher scores represent higher level of disability.

^c^ Range from 0 to 6; 3 items answered on a three point scale; higher scores represent higher level of support.

^d^ Range from 0 to 3; 3 items with a dichotomous response format (0 = no; 1 = yes); higher scores represent higher level of stress.

^e^ Range from 0 to 2; 2 items with a dichotomous response format (0 = no; 1 = yes); higher scores represent higher level of stress.

^f^ 1 = CIV; 2 = GHA.

^g^ SES: The median was used to differentiate between low (0) and high (1).

^h^ dichotomized: 0 = none formal education and primary education; 1 = secondary education and tertiary education

^j^ dichotomized: 0 = never married, separated, divorced or widowed; 1 = currently married or cohabiting

^k^ Wording of item: “Are there any other children of relatives/acquaintances in your household that you have to take care of?”

^l^ Apgar score after 1 minute: dichotomized: 0 = 5 to 8; 1 = 9 to 10.

^m^ 1 = male; 2 = female.

^n^ 0 = no; 1 = yes.

^p^ Number of unscheduled visits of women with women’s sick children.
